# The visibility of breastfeeding as a sexual and reproductive health right: a review of the relevant literature

**DOI:** 10.1186/s13006-022-00457-w

**Published:** 2022-03-05

**Authors:** Carina Stone, Julie P. Smith

**Affiliations:** 1 Tax and Transfer Policy Institute, Crawford School of Public Policy, Australian National University, Canberra, Australia; 2 College of Health and Medicine, Research School of Population Health, Australian National University, Canberra, Australia

**Keywords:** Breastfeeding, Visibility, Sexual and reproductive health rights, Gender responsive budgeting, Literature review

## Abstract

**Background:**

Governments must protect and apply maximum feasible resourcing to the protection, promotion and support of breastfeeding in order to meet their international legal obligations with respect to the human rights of women and children. However, governments across the world have consistently failed in these duties. Breastfeeding has been notably absent from mainstream feminist advocacy on sexual and reproductive health rights (‘SRH rights’). Why is there this lack of focus on breastfeeding in feminist advocacy in this area? This review seeks to identify the extent to which the protection, promotion and support of breastfeeding is visible within the SRH rights and the gender responsive budgeting literature.

**Method:**

A cross-disciplinary single scoping literature review of online and other databases was conducted to yield final samples of eighty-seven publications from the SRH rights literature and forty-four publications from the gender responsive budgeting literature. These publications were searched for references to breastfeeding.

**Results:**

Only 21% of the sexual and reproductive health rights literature and just one gender responsive budgeting publication sampled referenced the protection, promotion and support of breastfeeding. Where breastfeeding was mentioned in the publications reviewed it was, in general, brief and on the periphery of discussion.

**Conclusions:**

Reviews of the SRH rights literature and the gender budgeting literature both reveal an overwhelming absence of meaningful analysis on breastfeeding. The lack of attention to breastfeeding in the gender advocacy space represents a lost opportunity to advocate for the alleviation of the economic and social constraints imposed on breastfeeding women and caregivers.

**Supplementary Information:**

The online version contains supplementary material available at 10.1186/s13006-022-00457-w.

## Background

Breastfeeding is a recognised human right of women and children [1]. Yet, there continue to be egregious violations of these human rights with relation to breastfeeding, including throughout the COVID-19 pandemic. COVID-19 precautions have resulted in a proliferation of maternity care barriers to breastfeeding, including unnecessary separation of newborns from mothers, forced caesarean sections and prevention of breastfeeding in some cases, and inappropriate promotion of commercial food products for mothers and infants and young children through health channels [2–4]. This is in spite of authoritative World Health Organization (WHO) guidance emphasising that breastfeeding should be protected, supported and encouraged among all mothers including those with or at risk of COVID-19 due to its benefits to maternal and child health [5]. This paper will identify how breastfeeding rights fit into the broader sexual and reproductive health rights of women, and examine the visibility of breastfeeding within the sexual and reproductive health rights and gender responsive budgeting literature.

While the usual focus of health literature on the impacts of breastfeeding is its importance for children [6], breastfeeding is also important for women’s health and wellbeing [7, 8]. This is the case in both developed and developing country settings [9].

Breastfeeding is important for child spacing, accounting for many avoided pregnancies worldwide [9]. It has been estimated that 50% more births would be expected in the absence of breastfeeding in countries such as Uganda and Burkina Faso where continued breastfeeding is widespread [10]. This can be important for reducing reproductive stress from excessive pregnancies [11], as well as ameliorating economic, social, physical and emotional pressures on women and caregivers.

Research emphasises the importance of breastfeeding to other aspects of women’s physical health. A recent US study identified that suboptimal breastfeeding, defined in the study as breastfeeding that continues for less than the medical recommendations for exclusive breastfeeding for the first six months of life, with continued breastfeeding for at least one year, can be a risk factor for hypertension and myocardial infarction [12]. Systematic review research has shown that longer durations of breastfeeding are associated with lower risks of breast cancer, with potential protections against ovarian cancer and type 2 diabetes [9]. The scaling up of breastfeeding to near universal levels could prevent 20,000 annual deaths from breast cancer [9].

Breastfeeding is a recognised human right of both women and children embedded in several binding international human rights instruments [1]. This includes human rights in the context of women’s employment as well as their health and nutrition rights, and rights which are specific to pregnant and lactating mothers, and infants and young children [13, 14]. These human rights are enshrined in treaties including the United Nations *Convention on the Elimination of All Forms of Discrimination Against Women*, 1979 (CEDAW), the International Labour Organisation’s *Maternity Protection Convention*, 2000 (No. 183) (MPC), the United Nations *Convention on the Rights of the Child*, 1989 (CRC) and the *International Covenant on Economic, Social and Cultural Rights*, 1966. Table 1 provides a summary of the human rights instruments protecting breastfeeding. The process for treaties ratified by a State to become legally enforceable under domestic law depends according to the State, with two broad categories of monist or dualist legal systems. In monist jurisdictions, ratified treaties are immediately enforceable as domestic law. Under a dualist system, such as Australia, ratified treaties only become incorporated as part of domestic law when federal legislation is introduced. The United States system is a hybrid of these two approaches [15].

**Table 1:** Human rights instruments protecting breastfeeding

Year of adoption	Policy instrument (binding or non-binding)	Significance for breastfeeding
1919 (adopted by the International Labour Organisation)	Maternity Convention No. 3 (binding)	Article 3(d)- Two half-hour breaks to breastfeed during working hours. Articles 3(a), 3(b) and 3(c) provide for the payment of ‘benefits sufficient for the full and healthy maintenance of herself and her child’ for a maternity leave that covers six weeks before and six weeks after birth, as well as medical entitlements. Article 4- Unlawful to dismiss women during this maternity leave.
1948 (adopted by the UN General Assembly)	Universal Declaration of Human Rights (non-binding)	Article 25- ‘Everyone has the right to a standard of living adequate for the health and well-being of himself and of his family…Motherhood and childhood are entitled to special care and assistance.’
1952 (revised by the ILO)	Maternity Protection Convention No. 103 (binding)	Article 3- Maternity leave of at least 12 weeks, with extensions in the case of medical illness. Article 4- Compulsory social insurance to ensure cash benefits during maternity leave, and wider medical benefits. Article 6- Maternity leave dismissal unlawful. Article 5- Nursing breaks to be part of paid working hours.
1961	European Social Charter (binding)	Article 8- Maternity protections for employed women that include adequate social security benefits for a paid maternity leave of at least 12 weeks, unlawful dismissal during maternity leave, and ‘sufficient time off for’ mothers to nurse their infants.
1966 (adopted by the UN General Assembly)	International Covenant on Economic, Social and Cultural Rights (binding)	Article 10(2)- Special protection should be accorded to mothers during a reasonable period before and after childbirth. During such period working mothers should be accorded paid leave or leave with adequate social security benefits. Article 11(1)- The right of everyone to an adequate standard of living for himself and his family, including adequate food. Article 12(1)- 'The States Parties to the present Covenant recognize the right of everyone to the enjoyment of the highest attainable standard of physical and mental health.' Article 12(2)(a)- The provision for the reduction of the stillbirth-rate and of infant mortality and for the healthy development of the child.
1979 (adopted by the UN General Assembly)	Convention on the Elimination of All Forms of Discrimination against Women (CEDAW) (binding)	Article 10(h)- Access to specific educational information to help to ensure the health and well-being of families, including information and advice on family planning. Article 11(1)(f)- ‘The right to protection of health and to safety in working conditions, including the safeguarding of the function of reproduction.’ Article 11(2)(a) and (b)- ‘prohibit, subject to the imposition of sanctions, dismissal on the grounds of pregnancy or of maternity leave and discrimination in dismissals on the basis of marital status’; and require ‘maternity leave with pay or with comparable social benefits without loss of former employment, seniority or social allowances’. Article 12.2- States Parties shall ensure to women appropriate services in connection with pregnancy, confinement and the post-natal period, granting free services where necessary, as well as adequate nutrition during pregnancy and lactation.
1981 (adopted by the World Health Assembly)	International Code of Marketing of Breast-Milk Substitutes (non-binding) (‘The Code’)	Regulates and restricts infant formula marketing and other breastmilk substitutes to prevent interferences with and discouragement of breastfeeding.
1989 (adopted by the UN General Assembly) 1990 (Date in Force)	Convention on the Rights of the Child (binding)	Article 24(2)(e)- ‘To ensure that all segments of society, in particular parents and children, are informed, have access to education and are supported in the use of basic knowledge of child health and nutrition, the advantages of breastfeeding, hygiene and environmental sanitation and the prevention of accidents’. Article 24(2)(d)- To ensure appropriate pre-natal and post-natal health care for mothers. Article 18(2)- States Parties shall render appropriate assistance to parents and legal guardians in the performance of their child-rearing responsibilities and shall ensure the development of institutions, facilities and services for the care of children.
1990 (Organisation of African Unity)	African Charter on the Rights and Welfare of the Child (binding)	Article 2(h)- Repeats article 24(2)(e) of the CRC as above. Article 2(e)- To ensure appropriate health care for expectant and nursing mothers.
1990 (World Health Organisation and UNICEF)	Innocenti Declaration on the Protection, Promotion and Support of Breastfeeding. Breastfeeding in the 1990s: A Global Initiative (non-binding)	Stated that all women should be enabled to practise exclusive breastfeeding and all infants should be fed exclusively breastmilk up to 4-6 months of age, with continued breastfeeding following this. Call to action for implementation of initiatives to protect, promote and support breastfeeding, and to remove obstacles to breastfeeding.
1991 (World Health Organisation and UNICEF)	Baby-Friendly Hospital Initiative (BFHI) (non-binding)	Sets out ten steps for the protection, promotion and support of breastfeeding hospitals.
1995 (from the 4^th^ World Conference on Women)	Beijing Declaration and Platform for Action (non-binding)	Initiatives to protect, promote and support breastfeeding.
1999 (CEDAW Committee)	Interpretation (adopted as General Recommendation No. 24) of CEDAW requirements relating to Women and Health (Article 12)	No interpretative comments relating to breastfeeding.
2000 (revised by the ILO) 2002 (Date in force)	Maternity Protection Convention No. 183 (& Recommendation No. 191) (binding)	Article 4- Maternity Leave entitlements of not less than 14 weeks. Article 6- Cash benefits for maternity leave to be such that a woman can maintain herself and her child in proper conditions of health and with a suitable standard of living. Medical benefits to include postnatal care and hospitalisation care when necessary Article 8- Women cannot have their employment terminated on the basis of nursing. Women are guaranteed the right to return to the same position or an equivalent position paid at the same rate at the end of her maternity leave. Article 9- Maternity cannot be a source of discrimination in employment, including access to employment. Article 10- (1) A woman shall be provided with the right to one or more daily breaks or a daily reduction of hours of work to breastfeed her child. (2) The period during which nursing breaks or the reduction of daily hours of work are allowed, their number, the duration of nursing breaks and the procedures for the reduction of daily hours of work shall be determined by national law and practice. These breaks or the reduction of daily hours of work shall be counted as working time and remunerated accordingly. Recommendations No 191- 'frequency and length of nursing breaks should be adapted to particular needs’ on ‘production of medical certificates.’ ‘Where practicable and with the agreement of the employer and the woman concerned, it should be possible to combine the time allotted for daily nursing breaks to allow a reduction of hours of work at the beginning or at the end of the working day.’ ‘Where practicable, provision should be made for the establishment of facilities for nursing under adequate hygienic conditions at or near the workplace.’
2002 (endorsed by World Health Assembly)	WHO/UNICEF Global Strategy for Infant and Young Child Feeding (non-binding)	Sets out goals and initiatives for the protection, promotion and support of breastfeeding. Recommends exclusive breastfeeding for 6 months, and then up to two years or beyond with the ‘timely introduction of adequate and safe complementary foods’.
2003 (Assembly of the Union)	The Protocol to the African Charter on Human and People’s Rights on the Rights of Women in Africa (‘African Women’s Protocol’) (binding)	Article 14(2)(b)- State duties to establish and strengthen existing prenatal, delivery, and postnatal health and nutritional services for women during pregnancy and while they are breastfeeding. Article 24- Special Protection of Women in Distress- ensure the right of pregnant or nursing women or women in detention by providing them with an environment which is suitable to their condition and the right to be treated with dignity.
2005 (World Health Organisation and UNICEF)	Innocenti Declaration 2005 (non-binding)	Assessed progress since 1990, and called upon duty-holders to increase efforts to implement the targets in the 1990 Innocenti Declaration and the 2002 Global Strategy.
2013 (CRC Committee)	General Comment No. 15 on the right of the child to enjoy highest attainable standard of health	Noted the significant number of infant deaths caused by ‘suboptimal breastfeeding practices’, and called for ‘particular attention to ensuring full protection and promotion of breastfeeding practices’ in accordance with the WHO recommendations. Required states to implement and enforce the Code. Encouraged states to adopt the Baby-Friendly Hospital Initiative. Stated that private companies should comply with the Code.
2013 (CRC Committee)	General Comment No. 16 on State obligations regarding impact of business sector on children’s rights	Required the enforcement and implementation of the Code by states and relevant World Health Assembly resolutions. Requires states to create employment conditions within businesses that ‘support and facilitate breastfeeding’.
2016	Joint statement by the UN Special Rapporteurs on the Right to Food, Right to Health, the Working Group on Discrimination against Women in law and in practice, and the Committee on the Rights of the Child in support of increased efforts to promote, support and protect breast-feeding (non-binding)	Recognition of breastfeeding as a human right of the mother and the child, and calls for the end of inappropriate marketing of breast-milk substitutes. Calls for comprehensive and enforceable measures to promote, support and protect breastfeeding.

This table draws upon Table 1 of Galtry’s 2015 article on the human rights framework to protect breastfeeding [14].

Analyses of policy using the World Breastfeeding Trends Initiative (WBTi), an internationally recognised indicator, reveals a lack of funding at both country and global levels, for implementing the policies and programs known to better enable optimal breastfeeding [16]. These policies include paid maternity leave, quality maternity care and restraints on predatory marketing of breast milk substitutes [17–20]. This lack of funding is in spite of the obligations on governments to apply maximum feasible resourcing to realising rights such as measures enabling mothers and children to breastfeed [21]. Indeed, only 44 per cent of infants are breastfed exclusively for the first six months as per the World Health Organisation’s global recommendations [22]. The scaling up of breastfeeding rates to near universal levels could prevent 823,000 annual deaths in children younger than five years of age ([9], p475).

The lack of focus by policymakers and public health authorities on breastfeeding as a human right of women as well as children is troubling and harmful. This review seeks to identify the extent to which breastfeeding protection, promotion and support is visible within the SRH rights literature and the gender responsive budgeting literature.

Gender responsive budgeting can be defined as ‘an analysis of the impact of the budget on gender equality and a process of changing budgetary decision-making and priorities’ to improve gender equality outcomes ([23], p1). SRH rights has been described as encompassing ‘efforts to eliminate preventable maternal and neonatal mortality and morbidity, to ensure quality sexual and reproductive health services, including contraceptive services, and to address sexually transmitted infections (STI) and cervical cancer, violence against women and girls, and sexual and reproductive health needs of adolescents’ ([24], p30). To realise women’s and children’s human rights, governments must devote the maximum feasible resourcing needed to dismantle the ‘political, economic and sociocultural constraints in women’s lives that make breastfeeding difficult... (and) reduce women’s economic, political, and social status’ ([25], p3).

Literature from the above two particular fields of study were reviewed because these fields advocate for the legal protection of women’s SRH rights, the deployment of maximum feasible resourcing by governments to ensure these obligations are met, and the valuation and compensation of women’s unpaid labour [21]. These are all issues highly pertinent to the protection of mothers’ and children’s human rights with respect to breastfeeding. However, breastfeeding has been seen to be traditionally invisible within mainstream feminist advocacy [26]. Feminism has evolved over the last two decades, and feminist worldviews appear to be increasingly inclusive of breastfeeding. However, despite its impact on reproductive health and being a facet of reproductive autonomy, it is not clear whether breastfeeding is now more included in discussions of sexual and reproductive health rights in research or policy formulations. This paper therefore seeks to identify whether breastfeeding is visible in research articles on the topics of sexual and reproductive health rights and gender responsive budgeting.

### Research aims

Our study aims to assess the extent to which the protection, promotion and support of breastfeeding is visible within the SRH rights and the gender responsive budgeting literature.

## Methods

### Research design

Cross-disciplinary narrative literature searches were conducted to assess the visibility of breastfeeding firstly, within the SRH rights literature and secondly, in the gender responsive budgeting literature. The reviews of these two fields of study were conducted separately in order to obtain separate results as to what extent breastfeeding had entered the mainstream literature in the respective fields, particularly given that gender budgeting analysis is a newer field having originated in the 1980s [27]. No search terms specifically related to breastfeeding and infant feeding were included in order to review the visibility of breastfeeding within the general SRH rights and gender responsive budgeting literature.

The methods employed in this review were motivated to align with some features of the systematic review process. A specific research question, search strategy, and inclusion and exclusion criteria were defined in advance of the review. All citations that appeared in database searches using the search strategy were downloaded, and this sample was reduced through screening titles and abstracts on the basis of this pre-defined inclusion criteria, before a final stage of full-text review for eligibility. Some steps of the systematic review method, such as employing a Cochrane Risk of Bias tool, were not applied. Hence, this review has not been labelled a systematic review, but rather employs some of the features of the systematic review method in order to model some of the improved rigour, reproducibility and wide publication sourcing of the systematic review method. We consider this approach appropriate for this research project given the heterogeneous literature to be sampled and the motivation to explore the sociological context around breastfeeding, rather than a clinical purpose to evaluate the quantitative results of randomised control trials or qualitative data findings of a survey project.

### Setting and relevant context

Publications were only included if they were published in English, however no national or regional restrictions were applied.

### Sample: defining the articles reviewed

#### Sexual and Reproductive Health Rights Literature Review

Only English sources were included for both reviews for which full text copies were available online. Publications were excluded if they had a narrow focus on particular sexual and reproductive health rights issues, for example abortion or forced sterilisation. Publications were not excluded if they focused on sexual and reproductive health rights in specific contexts (for example disaster relief, HIV/AIDS issues, or internally displaced peoples), so long as the article intended to cover sexual and reproductive health rights as a whole.

#### Gender Responsive Budgeting Literature Review Methods

Publications were excluded after screening abstracts and titles if they focused on gender budgeting for specific outcomes unrelated to breastfeeding (for example girls’ education). Publications were also excluded if they focused on university gender budgets rather than government gender budgeting, given the focus in this article on maximum feasible resourcing by government. Finally, full text assessments excluded some publications where the focus was on the administrative and bureaucratic process of gender budgeting, with little to no detail on the substantive content of gender budgets or policy initiatives that they may be encouraging.

### Data collection: the search strategy and process

Journal articles, books and official reports published from 2003 onwards, after the publication of the WHO *Global Strategy for Infant and Young Child Feeding* (2003) (‘*The Global Strategy*’) [28], were included. *The Global Strategy*, which was endorsed by the World Health Assembly on the 18^th^ May 2002, recommended exclusive breastfeeding for six months, followed by continued breastfeeding for up to two years or beyond with safe and adequate complementary foods, setting up goals and initiatives to protect, promote and support this recommendation [28]. Given the significance of this report, only references published from 2003 were included in this review. The review searching was finalised in June 2020.

For the review of the SRH rights literature since 2003, electronic searches of various databases including ScienceDirect, SagePub, JSTOR, ProQuest and Google Scholar were conducted. Some sources were also included from hand searches to capture key references that may have been missed in the electronic searches. Search terms included “sexual and reproductive rights”, “reproductive rights”, “reproductive health”, “women’s rights”, “maternal rights”, “maternal health”, “Convention on the Elimination of All Forms of Discrimination Against Women”, the “Convention on the Rights of the Child”, and the “Maternity Protection Convention”.

For the review of the gender budgeting literature, electronic searches of databases including ScienceDirect, SagePub, JSTOR, ProQuest and Google Scholar, as well as some hand searches, identified publications. Given the smaller stock of gender budgeting literature compared to the SRH rights literature, more expansive search terms were used. Search terms included multiple variations of “gender budget”, “gender responsive budget” and “gender sensitive budget”.

### Measurement

Publications were searched for mentions of the terms ‘breastfeed’, ‘milk’, ‘lactation’, ‘nursing’, ‘infant feeding’, as well as their grammatical variations, within documents. Publications that did mention these terms in the context of breastfeeding were deemed to have referenced breastfeeding, with the content of these references noted. Action-oriented references to policies and practices that protect, promote and support breastfeeding were noted in a separate sub-category.

### Data analysis

There were eighty-seven publications included in the sample for review of the SRH rights literature. The citations for these SRH rights publications can be found in Additional file 1. There were forty-four publications included in the sample for review of the gender responsive budgeting literature. The citations for these gender responsive budgeting publications can be found in Additional file 2.

**Fig. 1:**
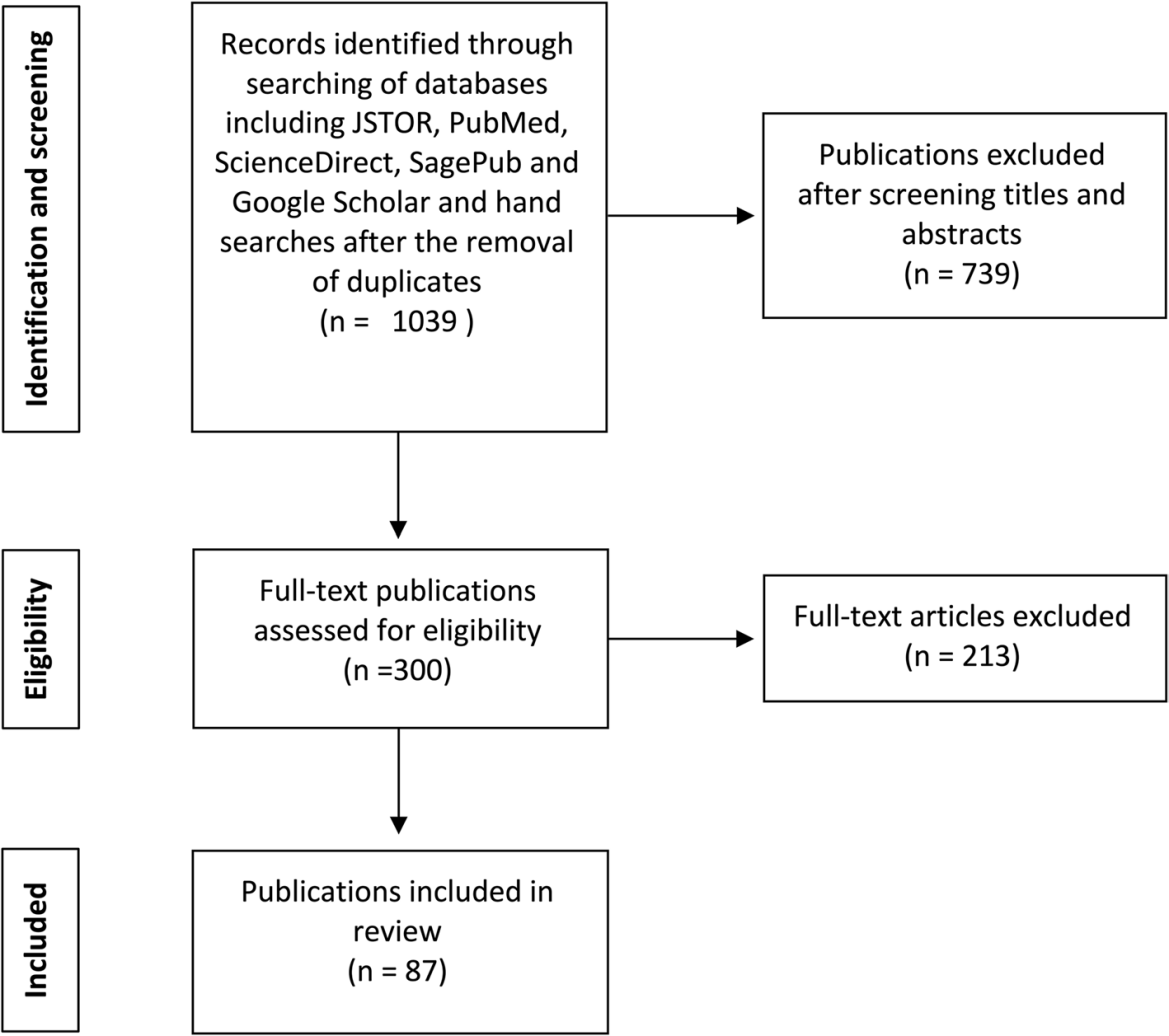
- PRISMA flow chart: sexual and reproductive health rights literature review

**Fig. 2:**
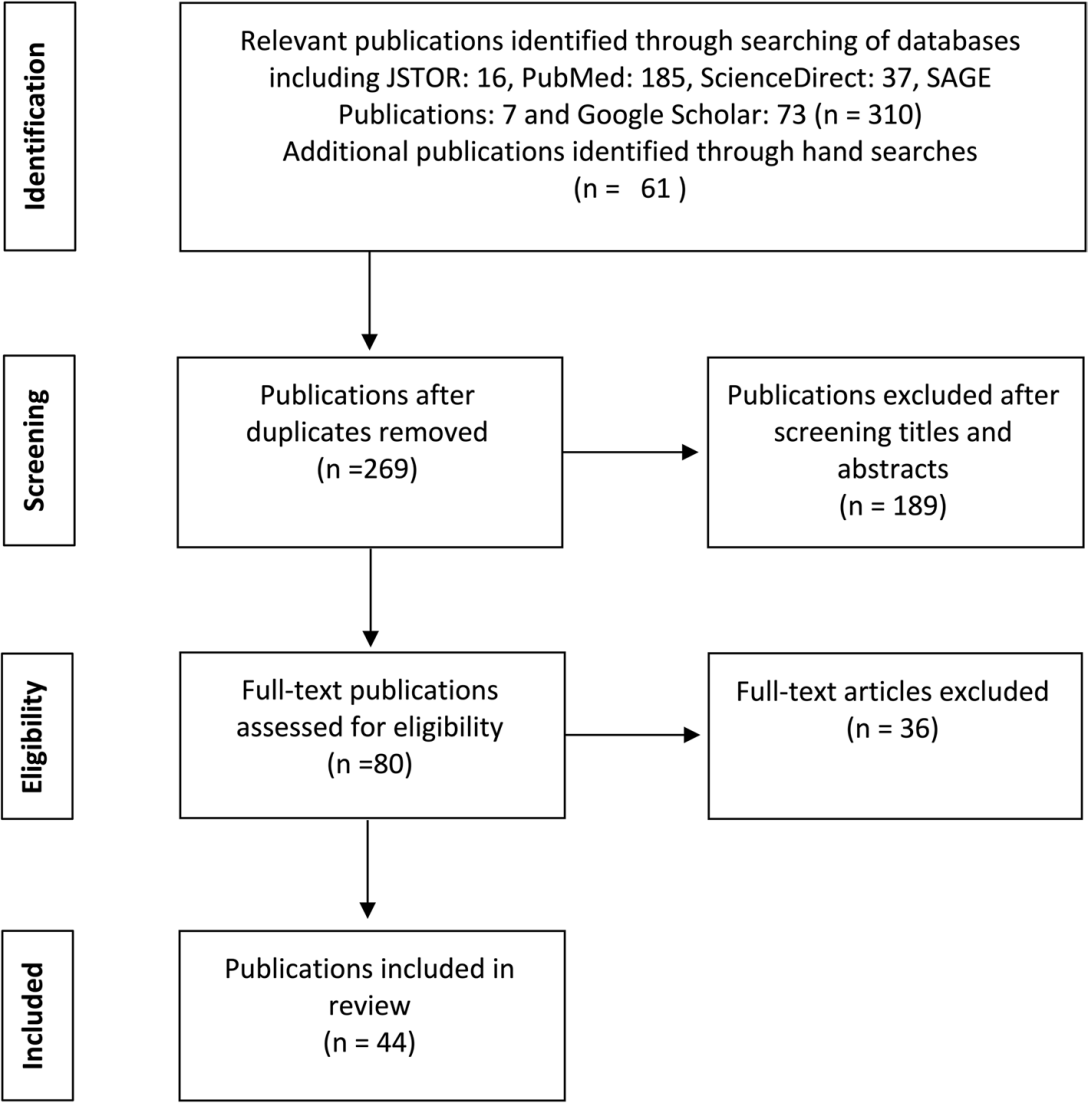
- PRISMA flow chart: gender responsive budgeting literature review

The PRISMA flow chart in Fig. 1 exhibits the number of records identified, included and excluded over the stages of the SRH rights review [29]. The PRISMA flow chart in Fig. 2 exhibits the number of records identified, included and excluded over the stages of the gender budgeting review [29].

## Results

Reviews of the SRH rights literature and the gender budgeting literature both reveal an absence of meaningful analysis on breastfeeding. Where breastfeeding was mentioned in the publications reviewed it was, in general, brief and on the periphery of discussion.

Most publications in the final sample for the SRH rights literature and the gender budgeting literature employed qualitative methods, eighty-four per cent and eighty-six per cent respectively. Publications covered a wide range of geographical areas of focus across the two reviews, including thirty-five different countries. Forty-four per cent of the total publications had a global focus. Publications in the SRH rights literature were more likely to narrow down into a particular sub-context or population of focus. Five of the eighty-seven SRH rights publications focused on women with a disability, five focused on emergency and conflict situations, four on HIV/AIDS, three on sexuality education, and two on women from a refugee background. The majority of publications were journal articles and books, at 93 per cent for the SRH rights review and 81 per cent for the gender budgeting review. Reports made up 7 per cent of the SRH rights review and 16 per cent of the gender budgeting review samples.

### SRH rights literature review results

**Fig. 3:**
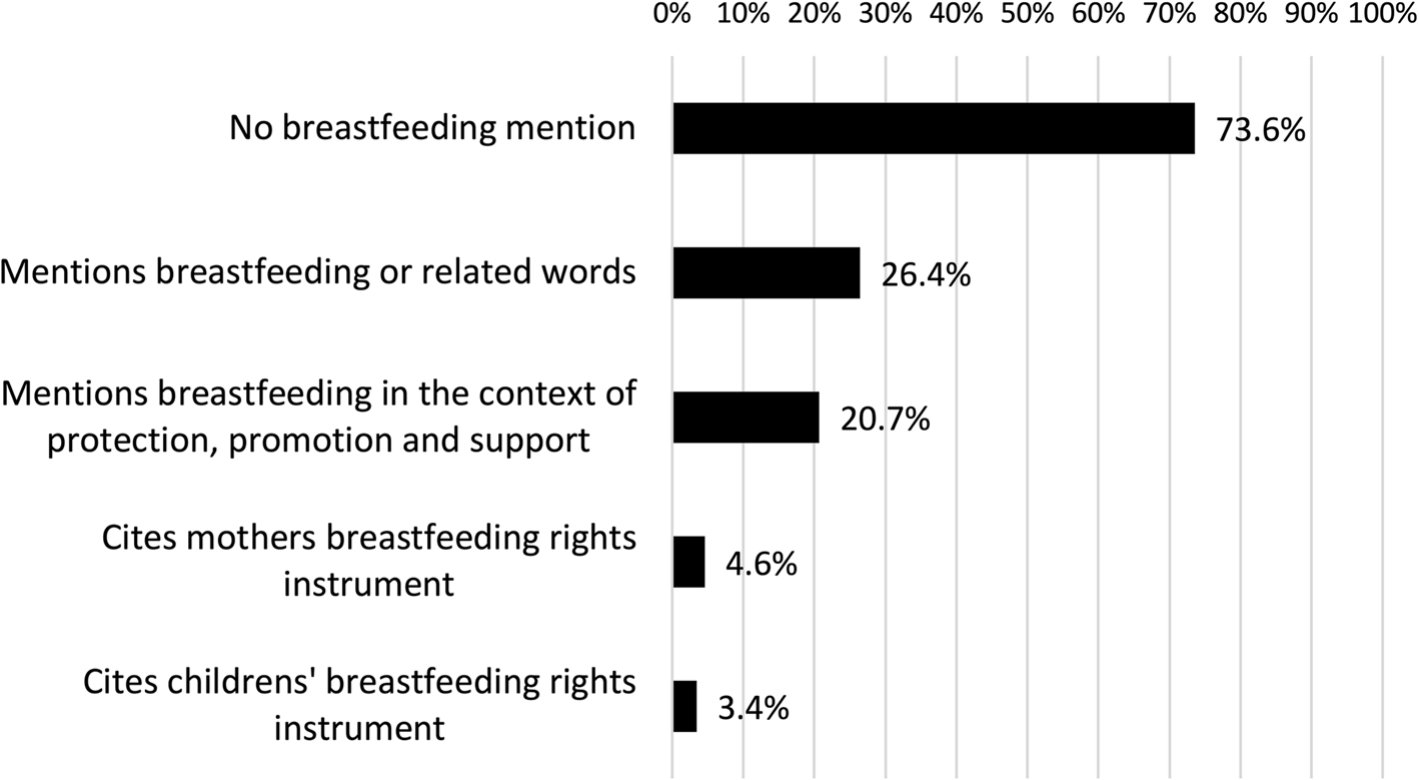
- The visibility of breastfeeding in the sexual and reproductive health rights literature (*n* = 87)

Figure 3 demonstrates over 70 per cent of SRH rights articles reviewed made no reference to breastfeeding. Of the eighty-seven publications reviewed, sixty-four papers were found to have zero references to breastfeeding and its related terms. Several publications with a focus on maternity and maternal health failed to mention breastfeeding even once [30–32].

Twenty-three publications were found to have some reference to breastfeeding and related terms, and eighteen with references to breastfeeding protection, promotion and support. Even among these studies, meaningful and detailed discussion of breastfeeding was minimal. The content of references to breastfeeding in multiple publications were by referring to national, regional or international legal instruments or definitions of reproductive health that include breastfeeding, but did not otherwise comment on breastfeeding [33–37]. Furthermore, three papers explicitly referenced a children’s rights instrument on breastfeeding, including the *Convention on the Rights of the Child* (1989) and the *African Charter on the Rights and Welfare of the Child* (1990) [14, 38, 39]. Four articles explicitly referenced a women’s rights instrument on breastfeeding, particularly Articles 14 and 24 of the *Protocol to the African Charter on Human and Peoples’ Rights on the Right of Women in Africa* (2003) and Article 12 of the *Convention on the Elimination of Discrimination Against Women* (1979) [14, 35, 37, 39].

Figure 3 summarises these results. Additional file 3 provides a summary of the content of the references to breastfeeding in the twenty-three publications found to have mentioned breastfeeding.

### Gender responsive budgeting literature review results

**Fig. 4:**
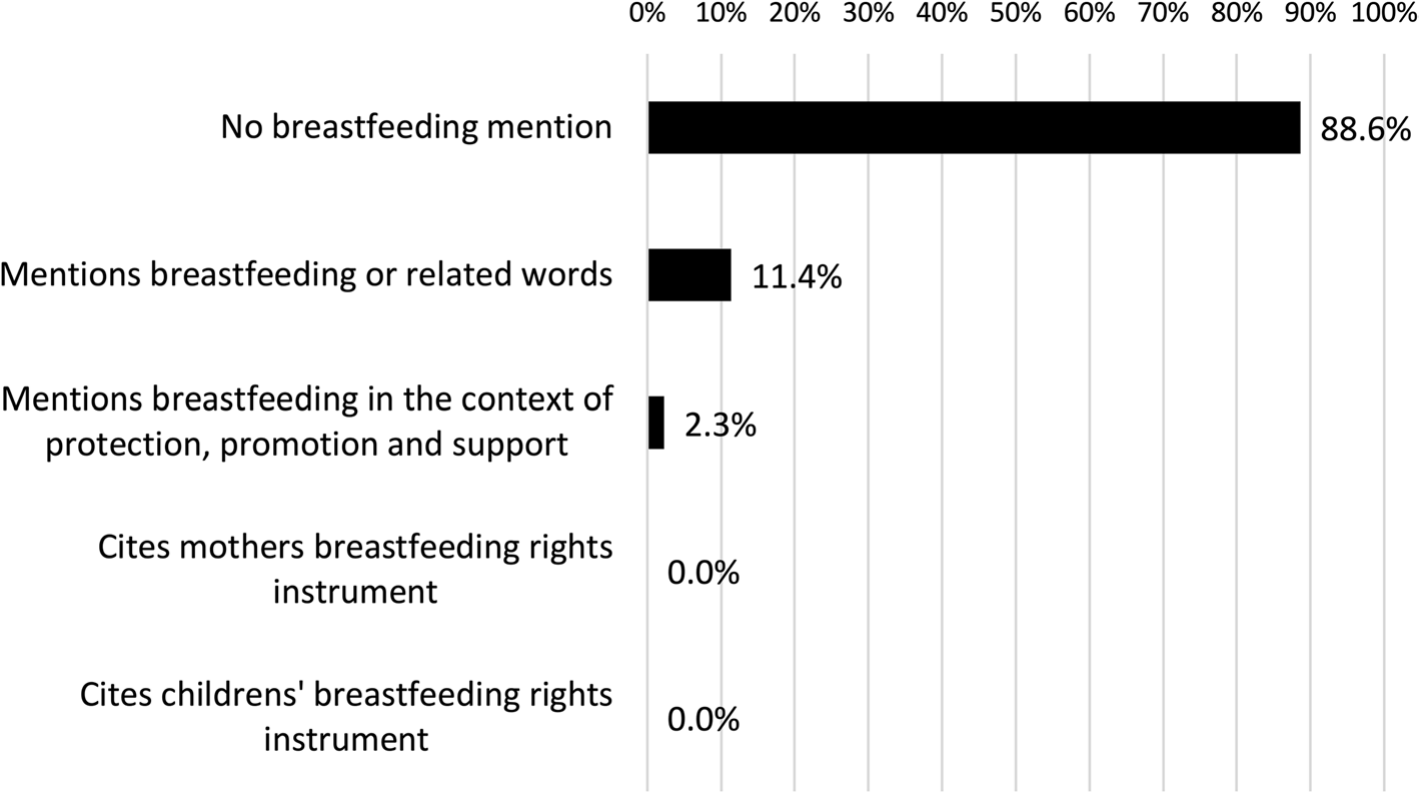
- The visibility of breastfeeding in the gender response budgeting literature (*n* = 44)

Figure 4 demonstrates that over 85 per cent of gender responsive budgeting articles reviewed made no reference to breastfeeding. Of the forty-four publications reviewed on gender budgeting, thirty-nine had zero mentions of breastfeeding and related terms. Additional file 4 provides a summary of the content of the references to breastfeeding in the five publications found to have mentioned breastfeeding.

Most publications on gender budgeting analysed the importance of gender budgets in recognising, valuing and integrating into government budgeting the ‘positive externality to others in the household or society at large’ of women and girls’ unpaid household and care work ([40], p33). However, only one publication recognised breastfeeding as an aspect of this unpaid care work amongst the forty-four publications [41].

Of the five publications that referenced breastfeeding, none made detailed comments. Only one of these publications made a reference to the protection, promotion or support of breastfeeding [42]. Three of the five publications that referenced breastfeeding were in the context of the provision of supplementary nutritious food to nursing mothers as part of the Anganwadi centres (child health centres) in India [43–45].

## Discussion

Our analysis has shown a distinct invisibility of breastfeeding in the literature on SRH rights and gender responsive budgeting. Where it was considered at all, emphasis was given to children’s rights to breastfeeding, HIV transmission and breastfeeding, breastfeeding in emergencies and disasters, or token reference to international documents or definitions stating the need for breastfeeding protection, promotion and support. This finding is consistent with comments on the paucity of literature in other publications (e.g., [46]), and the lack of consideration of breastfeeding within feminist advocacy and gender equality legal protections is specifically explored in some publications (e.g., [14, 47]).

This relative invisibility is despite the importance of breastfeeding to both the reproductive health of women and to the exercise of reproductive autonomy following the birth of children. Approaching breastfeeding from a rights framework is important given that such a framework highlights the ‘structural elements influencing personal decisions and practices’ ([48], p19).

### Why has breastfeeding been absent from the SRH rights and gender responsive budgeting agendas?

We suggest that the invisibility of breastfeeding in the SRH rights and gender responsive budgeting literature reflects four main factors.

Firstly, a major driver of the SRH rights movement has historically been the population control agenda for which abortion and contraceptive access were central, and not necessarily reproductive health rights and reproductive autonomy [49]. Relatedly it is worthwhile reflecting on the differences we found between the different publication types. Reports were typically commissioned by international intergovernmental bodies and were more likely to have a programmatic policy or health discipline focus, whereas academic publications were more likely to reflect a feminist sociological approach of policy critique. One might expect that the reason for the invisibility of breastfeeding differs between these two formats. International reports perhaps avoid delving deeply into breastfeeding due to a potential political sensitivity about promoting child spacing as a sexual and reproductive health right, whereas the academic articles may not be as grounded in (what may be seen as) public health issues rather than social science issues. The consequence of excluding breastfeeding from academic research articles is that a format that typically focuses on holding policy makers accountable for accelerating gender equality issues does not include breastfeeding as part of this critique. Alternatively, the invisibility of breastfeeding in technical reports is concerning as it suggests that breastfeeding is not being adequately considered in policy formulation.

Secondly and crucially, exclusion of breastfeeding from gender equality agendas may reflect the consideration of breastfeeding as being exclusively in the interests of the child and hence only the child’s right. This reflects the lack of a recognition of an interdependent mother–child feeding dyad relationship within what is the dominant individual liberal rights frameworks of many national and international legal systems ([46], p1118).

This is reinforced by the narrowing of the discussion on breastfeeding to a programmatic public health focus and away from a strategic human rights focus ([46], p1102). Key institutional bodies such as UNICEF have adopted, in response to the demand for quantifiable health improvements, ‘operational, target-based approaches’ rather than framing and pursuing breastfeeding policies under the banner of human rights ([46], p1102). Meier and Labbok have reflected that ‘the absence of a scholarly foundation for human rights in breastfeeding policy’ has limited the development of international legal human rights protections from the harms of inappropriate breast milk substitute marketing and lobbying in the developing world ([46], p1075). A public health focus limits the focus to children’s wellbeing given the long-time deficiency of research into the maternal health importance of breastfeeding. Indeed, one recent publication on gender and health published in *The Lancet*, in inquiring as to the cause of observed health improvements, stated plainly that there lacked research on ‘whether these improvements occur entirely through mechanisms unrelated to gender equality, such as facilitating breastfeeding’ ([50], p2526]. This statement inferring that breastfeeding and gender equality are unrelated is in utter conflict with the literature and evidence that many women cannot breastfeed due to gender equality related barriers such as a lack of paid maternity leave, parent-friendly workplaces, etc. [47]. The invisibility of breastfeeding in sexual and reproductive health rights and gender responsive budgeting literature likely reflects therefore the categorisation of breastfeeding as a public health issue rather than a gender issue.

Thirdly and relatedly, within feminist movements ‘pressure to breastfeed in order to ensure infant health’ ([48], p16), particularly in contexts where a lack of support leaves women materially disadvantaged by breastfeeding, has been perceived as ‘part of a larger cultural project to maintain patterns of female domesticity and subjugation’ ([48], p16). This is heightened by a long history of maternal reproductive traits being used to justify the subordination of women and their exclusion from the public sphere. This highlights the importance of ensuring that women are adequately supported and compensated for breastfeeding through policies including adequate paid maternity and parental leave, and in removing both direct and indirect discrimination in the workplace of people who take on caregiving roles.

Finally, the growing commercial milk formula (CMF) industry have used lobbying to weaken national breastfeeding protection legislation in several countries, including lobbying in the US by CMF companies prior to a World Health Assembly meeting in 2018 [51, 52]. Formula has been consistently marketed as ‘liberation in a can’ that empowers women to work and be freed from the constraints of domestic obligations, with breastfeeding represented as the realm of traditionalists rather than women in the labour force [14, 53]. The ‘rhetorical link’ made by formula marketing between women’s freedom and formula use is predicated on a legal approach of only pursuing ‘formal equality’, rather than the more robust protections of ‘substantive equality’, such that the structure of market work is not being reformed to accommodate care work such as breastfeeding, through maternity leave and flexible working policies ([54], p11, [55]). Breast milk substitutes marketing, such as in the Similac ‘Sisterhood of Motherhood’ advertisement [52], have framed the public narrative as a discourse on lifestyle in a manner that suppresses scientific discussion of breastfeeding and opens a moral narrative on infant and young child feeding decisions that provokes judgment of parents who face structural barriers that in fact deny them a choice to breastfeed [54]. Over US$25 million was spent advertising infant formula and toddler milk in the USA in 2015 [52], and recent research has highlighted the political power of the industry to achieve and maintain policies that suit their commercial interests [56]

### Limitations

A limitation of this study is that it identified studies from across a number of disciplines in order to sample the wide literature and determine the overall visibility of breastfeeding from this literature. As a result, the identified literature was highly heterogeneous in both methodology and focus. Additionally, the study did not separate out the different types of publications (reports vs journal articles and books) to distinguish the results from the analysis of research articles as compared to reports. This could have provided more nuanced results about the global politics of population control and sensitivities on the child spacing effects of breastfeeding, and warrants further consideration in future research.

In addition, the exclusion of publications not published in English limits the cultural and territorial scope of the literature reviewed.

This study identifies a need for future research to understand how gender impact analysis, sexual and reproductive health rights advocacy and gender responsive budgeting can be applied to the breastfeeding policy area to dismantle the substantial economic and social costs that women currently face when breastfeeding [57], and enable their human rights. The protection, promotion and support of breastfeeding is all the more important during emergencies where women’s rights to reproductive bodily autonomy, especially to breastfeed, or to refuse caesarean section or separation from the newborn, are abandoned under pressure. One hundred and sixteen million babies are expected to have been born in the 40 weeks after COVID-19 was recognised as a pandemic on March 11 2020 [58]. The long-standing inadequate enforcement, funding and implementation of human rights to breastfeed around the world have meant that during this pandemic, 116 million birthing women have potentially had these health rights undermined.

## Conclusions

This study finds that meaningful discussion of breastfeeding is overwhelmingly absent from the sexual and reproductive health rights literature, despite several key international human rights instruments protecting women’s rights on breastfeeding. Equally, breastfeeding is almost invisible in the gender responsive budgeting literature that aims to value and compensate women’s unpaid labour. This is despite breastfeeding being an archetypical example of the discrimination women and parents face when they have a baby, and the financial disadvantages of providing unpaid care labour.

The lack of attention to breastfeeding in the gender advocacy space is concerning, as it represents a lost opportunity to advocate for the alleviation of the economic and social constraints on women who breastfeed, as well as ensure the social support needed to fulfil the reproductive autonomy of mothers and caregivers who want their child to be breastfed. Equally, for public health advocates a gendered approach to breastfeeding is essential to raise optimal breastfeeding rates, given that institutional structures that devalue care work and breastfeeding and hence provide inadequate support are a major reason for introducing breast milk substitutes [59].

The wellbeing, autonomy and health of women as well as infants and their families around the world depend on addressing these constraints. As the UN Development Report stated in 1999,

*“Human support to others is essential for social cohesion and a strong community. It is also essential for economic growth. But the market gives few incentives and few rewards for it...Families, nations and corporations have been free-riding on caring labour provided mostly by women, unpaid or underpaid” *([60], p7)*.*

Gender bias within current economic statistical systems has led to the invisibility within macroeconomic frameworks of unpaid household labour despite its significant economic contributions, something that gender responsive budgeting seeks to highlight. Within the health economic literature, unpaid care work of breastfeeding is notably invisible ([61], p480), despite, according to one major study ‘not breastfeeding [being] associated with... economic losses of about $302 billion annually’ ([17], p491). Breastfeeding remains consistently invisible within reproductive and caring labour despite its important economic and social contributions.

Whilst many women and parents aspire to exclusively breastfeed their child as per the WHO recommendations, the economic and social disadvantages caused by lack of social and financial support by government and society mean many are unable to fulfil this desire [62]. Marginalised, low-income mothers are most likely to be employed in work that does not support breastfeeding, and due to racial differences in employment patterns women of colour, and their infants, are particularly impacted by the lack of structural support for breastfeeding [63]. This represents a clear example of interference with reproductive and bodily autonomy. Hence the protection, promotion and support of breastfeeding and the interdependent mother-infant feeding dyad must be prioritised by human rights frameworks, and the need for funding of supportive policies and programs must be highlighted in future gender budgeting analyses.

## Supplementary Information

**Additional file 1:** Sexual and reproductive health rights literature review sample. This file includes the citation for each publication included in the sample for the review of the sexual and reproductive health rights literature. It also includes a brief note of each article’s geographical focus, sub-context and method.

**Additional file 2:** Gender responsive budgeting literature review sample. This file includes the citations for each publication included in the sample for the review of the gender responsive budgeting literature. It also includes a brief note of each article’s geographical focus, sub-context and method.

**Additional file 3:** References to breastfeeding in the sexual and reproductive health rights literature review. This file includes the citations for the publications which referred to breastfeeding in the sexual and reproductive health rights literature review. These publications are separated into, firstly, those that contained references to breastfeeding which were not deemed to be about the protection, promotion or support of breastfeeding, and secondly publications that did contain references to the protection, promotion or support of breastfeeding. A summary of the context of the references to breastfeeding are included for each publication.

**Additional file 4:** References to breastfeeding in the gender responsive budgeting literature review. This file includes the citations for the publications which referred to breastfeeding in the gender responsive budgeting literature review. These publications are separated into, firstly, those that contained references to breastfeeding which were not deemed to be about the protection, promotion or support of breastfeeding, and secondly publications that did contain references to the protection, promotion or support of breastfeeding. A summary of the context of the references to breastfeeding are included for each publication.

## Data Availability

Not applicable.
