# Supplementary material for: The visibility of breastfeeding as a sexual and reproductive health right: a review of the relevant literature
Source: Int Breastfeed J. 2022 Mar 5;17:18. doi: 10.1186/s13006-022-00457-w (PMC8897768; doi:10.1186/s13006-022-00457-w)
Supplement: Supplementary file 1 — Additional file 1: Sexual and reproductive health rights literature review sample. This file includes the citation for each publication included in the sample for the review of the sexual and reproductive health rights literature. It also includes a brief note of each article’s geographical focus, sub-context and method. [file 13006_2022_457_MOESM1_ESM.docx]

*Sexual and reproductive health rights literature review sample*

| Sample Publications | Geographical focus | Sub-context | Method |
| --- | --- | --- | --- |
| Addlakha R, Price J, Heidari S. Disability and sexuality: claiming sexual and reproductive rights. Reprod Health Matters. 2017;25(50):4-9. https://doi.org/10.1080/09688080.2017.1336375. | Global | Disability | Qualitative |
| Alburo-Cañete KZK. Bodies at risk: “managing” sexuality and reproduction in the aftermath of disaster in the Philippines. Gend Technol Dev. 2014;18(1):33-51. https://doi.org/10.1177/0971852413515356. | Philippines | Emergency | Qualitative |
| Alzate MM. The sexual and reproductive rights of internally displaced women: the embodiment of Colombia's crisis. Disasters. 2008;32(1):131-48. https://doi.org/10.1111/j.1467-7717.2007.01031.x. | Colombia | Emergency | Qualitative |
| Alzate MM. The role of sexual and reproductive rights in social work practice. Affilia. 2009;24(2):108-19. https://doi.org/10.1177%2F0886109909331695. | Global | Social work | Qualitative |
| Amroussia N, Goicolea I, Hernandez A. Reproductive health policy in Tunisia: women's right to reproductive health and gender empowerment. Health Hum Rights 2016;18(2):183-94. https://pubmed.ncbi.nlm.nih.gov/28559685/. | Tunisia |  | Qualitative |
| Bakhru TS. Reproductive health and human rights: lessons from Ireland. J Int Women's Stud. 2017;18(2):27-44. https://vc.bridgew.edu/jiws/vol18/iss2/2/. | Ireland |  | Qualitatitive |
| Balogun V, Durojaye E. The African Commission on Human and Peoples' Rights and the promotion and protection of sexual and reproductive rights. Afr Hum Rights Law J. 2011;11(2):368-95. | Africa |  | Qualitative |
| Barroso C, Sippel S. Sexual and reproductive health and rights: integration as a holistic and rights-based response to HIV/AIDS. Women's Health Issues. 2011;21(6, Supplement):250-4. https://doi.org/10.1016/j.whi.2011.07.002. | Global | HIV/AIDS | Qualitative |
| Bayeh E. The role of empowering women and achieving gender equality to the sustainable development of Ethiopia. Pacific Science Review B: Humanities and Social Sciences. 2016;2(1):37-42. https://doi.org/10.1016/j.psrb.2016.09.013. | Ethiopia |  | Qualitative |
| Bedford K. Care and the 53rd commission on the status of women: a transformative policy space? Reprod Health Matters. 2011;19(38):197-207. https://doi.org/10.1016/S0968-8080(11)38576-X. | Global | Care | Qualitative |
| Bennett B. Globalising the body: globalisation and reproductive rights. UNSW Law Journal. 2006;29(2):266-71. | Global |  | Qualitative |
| Bernstein S. Are sexual and reproductive rights only for the rich? Development. 2005;48(4):99-105. https://doi.org/10.1057/palgrave.development.1100185. | Global |  | Qualitative |
| Berro Pizzarossa L. Here to stay: The evolution of sexual and reproductive health and rights in International Human Rights Law. Laws. 2018;7(3):29. https://doi.org/10.3390/laws7030029. | Global |  | Qualitative |
| Bonder G, Radjavi M, Ramirez C. Advancing women's sexual and reproductive rights in Argentina : challenges to implementing international law. Can Wom Studies. 2009;27(1):121-31. https://cws.journals.yorku.ca/index.php/cws/article/view/23152/21431. | Argentina |  | Qualitative |
| Briozzo L, Faúndes A. The medical profession and the defense and promotion of sexual and reproductive rights. Int J Gynaecol Obstet. 2007;100(3):291-4. https://doi.org/10.1016/j.ijgo.2007.09.015. | Global |  | Qualitative |
| Center for Reproductive Rights U. ICPD and human rights: 20 years of advancing reproductive rights through UN treaty bodies and legal reform. New York: Center for Reproductive Rights; 2013. https://www.unfpa.org/publications/icpd-and-human-rights. Accessed 8 October 2020. | Global |  | Qualitative |
| Chaudhuri S. A life course model of human rights realization, female empowerment, and gender inequality in India. World Dev. 2013;52:55-70. http://doi.org/10.1016/j.worlddev.2013.07.001. | India |  | Quantitative |
| Cook RJ, Dickens BM, Fathalla MF. Reproductive health and human rights: integrating medicine, ethics, and law. Oxford; Clarendon Press; 2003. | Global |  | Qualitative |
| Danielsson M, Sundström K. Chapter 6: reproductive health. Scand J Public Health. 2006;34(67_suppl):147-64. http://doi.org/10.1080/14034950600677204. | Sweden |  | Qualitative |
| Davis MF, Withers B. Reproductive rights in the legal academy: a new role for transnational law. J Legal Educ. 2009;59(1):35-59. | United States | Legal education | Qualitative |
| Devine A, Ignacio R, Prenter K, Temminghoff L, Gill-Atkinson L, Zayas J, et al. “Freedom to go where I want”: improving access to sexual and reproductive health for women with disabilities in the Philippines. Reprod Health Matters. 2017;25(50):55-65. https://doi.org/10.1080/09688080.2017.1319732. | Philippines | Disability | Qualitative |
| Djafri D, Chongsuvivatwong V, Geater A. Effect of the September 2009 Sumatra earthquake on reproductive health services and MDG 5 in the city of Padang, Indonesia. Asia Pac J Public Health. 2015;27(2):NP1444-NP56. https://doi.org/10.1177/1010539513496841. | Indonesia | Emergency | Quantitative |
| Dos Santos SB. Controlling black women’s reproductive health rights: An impetus to black women’s collective organizing. Cult Dyn. 2012;24(1):13-30. https://doi.org/10.1177/0921374012452809. | Brazil | Black women’s movement | Qualitative |
| Erdman JN, Cook RJ. Women’s rights to reproductive and sexual health in a global context. J Obstet Gynaecol Can. 2006;28(11):991-7. https://doi.org/10.1016/S1701-2163(16)32295-2. | Global |  | Qualitative |
| Friedman EJ. Gendering the agenda: the impact of the transnational women's rights movement at the UN conferences of the 1990s. Womens Stud Int Forum. 2003;26(4):313-31. https://doi.org/10.1016/S0277-5395(03)00077-3. | Global |  | Qualitative |
| Galtry J. Strengthening the human rights framework to protect breastfeeding: a focus on CEDAW. Int Breastfeed J. 2015;10:29. https://doi.org/10.1186/s13006-015-0054-5. | Global |  | Qualitative |
| Germain A, Sen G, Garcia-Moreno C, Shankar M. Advancing sexual and reproductive health and rights in low-and middle-income countries: implications for the post-2015 global development agenda. Glob Public Health. 2015;10(2):137-48. https://doi.org/10.1080/17441692.2014.986177. | Global |  | Qualitative |
| Gerntholtz L, Gibbs A, Willan S. The African women's protocol: bringing attention to reproductive rights and the MDGs. PLoS Med. 2011;8(4):e1000429. http://dx.doi.org/10.1371/journal.pmed.1000429. | Africa |  | Qualitative |
| Gianella C, Yamin AE. Struggle and resistance: using international bodies to advance sexual and reproductive rights in Peru. Berkeley J Gender L & Just. 2018;33(1):41-73. http://dx.doi.org/10.15779/Z38Z31NP3Z. | Peru |  | Qualitative |
| Girard F. Taking ICPD beyond 2015: negotiating sexual and reproductive rights in the next development agenda. Glob Public Health. 2014;9(6):607-19. https://doi.org/10.1080/17441692.2014.917381. | Global |  | Qualitative |
| Girard F. Implications of the Trump administration for sexual and reproductive rights globally. Reprod Health Matters. 2017;25(49):6-13. https://doi.org/10.1080/09688080.2017.1301028. | Global |  | Qualitative |
| Guerin PB, Allotey P, Elmi FH, Baho S. Advocacy as a means to an end: assisting refugee women to take control of their reproductive health needs. Women Health. 2006;43(4):7-25. https://doi.org/10.1300/j013v43n04_02. | Australia and New Zealand | Refugee women | Mixed |
| Gupta GR, Oomman N, Grown C, Conn K, Hawkes S, Shawar YR, et al. Gender equality and gender norms: framing the opportunities for health. Lancet. 2019;393(10190):2550-62. https://doi.org/10.1016/S0140-6736(19)30651-8. | Global |  | Qualitative |
| Haslegrave M. Integrating sexual and reproductive rights into the medical curriculum. Best Pract Res Clin Obstet Gynaecol. 2006;20(3):433-45. https://doi.org/10.1016/j.bpobgyn.2006.01.002. | Global | Medical curriculum | Qualitative |
| Hill BJ. Reproductive rights as health care rights. Colum J Gender & L. 2008;18(2):501-50. https://doi.org/10.7916/cjgl.v18i2.2570. | United States, South Africa and Canada |  | Qualitative |
| Hunt X, Carew MT, Braathen SH, Swartz L, Chiwaula M, Rohleder P. The sexual and reproductive rights and benefit derived from sexual and reproductive health services of people with physical disabilities in South Africa: beliefs of non-disabled people. Reprod Health Matters. 2017;25(50):66-79. http://doi.org/10.1080/09688080.2017.1332949. | South Africa | Disability | Quantitative |
| International Planned Parenthood Foundation. IPPF framework for comprehensive sexuality education (CSE). London: IPPF; 2010. https://www.ippf.org/sites/default/files/ippf_framework_for_comprehensive_sexuality_education.pdf. Accessed 14 September 2020. | Global | Sexuality education | Qualitative |
| Karam A. Positions on sexual and reproductive rights in Muslim-majority countries and institutions: a telling indication of things to come? Dev Pract. 2017;27(5):698-707. https://doi.org/10.1080/09614524.2017.1327025. | Muslim-majority countries |  | Qualitative |
| Khanna A, Pradhan J, Harun-Ar-Rashid, Beekink E, Gupta M, Sharma A. Financing reproductive health in Bangladesh. J Health Manag. 2013;15(2):177-201. https://doi.org/10.1177/0972063413489004. | Bangladesh |  | Mixed |
| Kismödi E, Ferguson L. Celebrating the 70th anniversary of the UDHR, celebrating sexual and reproductive rights. Reprod Health Matters. 2018;26(52):1-5. http://doi.org/10.1080/09688080.2018.1550239. | Global |  | Qualitative |
| Koenen KC, Lincoln A, Appleton A. Women's status and child well-being: A state-level analysis. Soc Sci Med. 2006;63(12):2999-3012. https://doi.org/10.1016/j.socscimed.2006.07.013. | United States |  | Quantitative |
| Kuhlik L. Pregnancy behind bars: the constitutional argument for reproductive healthcare access in prison. Harv CR-CLL Rev. 2017;52:501. | United States | Women in prison | Qualitative |
| Kulczycki A. Ethics, ideology, and reproductive health policy in the United States. Stud Fam Plan. 2007;38(4):333-51. https://doi.org/10.1111/j.1728-4465.2007.00145.x. | United States |  | Qualitative |
| Kwadwo W, Anafi P, Sekyere FO. Does disability matter? Disability in sexual and reproductive health policies and research in Ghana. Int Q Community Health Educ. 2014;35(1):21-35. https://doi.org/10.2190/IQ.35.1.c. | Ghana | Disability | Qualitative |
| Laporta Hernández E. Legal strategies to protect sexual and reproductive health and rights in the context of the refugee crisis in Europe: a complaint before the European Ombudsperson. Reprod Health Matters. 2017;25(51):151-60. https://doi.org/10.1080/09688080.2017.1405675. | Europe | Refugee women | Qualitative |
| Lee E-KO, Oh H. A wise wife and good mother: reproductive health and maternity among women with disability in South Korea. Sex Disabil. 2005;23(3):121-44. http://doi.org/10.1007/s11195-005-6728-y. | South Korea | Disability | Quantitative |
| Lopreite D. Gender policies in Argentina after neoliberalism: opportunities and obstacles for women's rights. Lat Am Perspect. 2015;42(1):64-73. http://dx.doi.org/10.1177/0094582X13492709. | Argentina |  | Qualitative |
| Mehta S. The AIDS pandemic: A catalyst for women's rights. Int J Gynaecol Obstet. 2006;94(3):317-24. https://doi.org/10.1016/j.ijgo.2006.04.020. | Global | HIV/AIDS | Qualitative |
| Mocumbi P, Amaral E. Reproductive rights and HIV/AIDS. Best Pract Res Clin Obstet Gynaecol. 2006;20(3):381-93. https://doi.org/10.1016/j.bpobgyn.2006.01.004. | Global | HIV/AIDS | Qualitative |
| Morgan LM. Reproductive rights or reproductive justice? Lessons from Argentina. Health Hum Rights 2015;17(1):136-47. | Argentina |  | Qualitative |
| Nair S, Sexton S, Kirbat P. A decade after Cairo: women's health in a free market economy. Indian J Gend Stud. 2006;13(2):171-93. https://doi.org/10.1177/097152150601300203. | Global |  | Qualitative |
| Ngwena CG, Brookman-Amissah E, Skuster P. Human rights advances in women’s reproductive health in Africa. Int J Gynaecol Obstet. 2015;129(2):184-7. https://doi.org/10.1016/j.ijgo.2015.02.001. | Africa |  | Qualitative |
| O'Connell C. Litigating reproductive health right in the inter-American system: what does a winning case look like? Health Hum Rights 2014;16(2):116-28. https://dx.doi.org/10.2139/ssrn.2553649. | Inter-America |  | Qualitative |
| Orza L, Crone T, Mellin J, Westerhof N, Stackpool-Moore L, Restoy E, et al. Searching for the second R in sexual and reproductive health and … rights. J Adolesc Health. 2017;60(2, Supplement 2):10-4. https://doi.org/10.1016/j.jadohealth.2016.11.006. | Global |  | Qualitative |
| Petchesky RP. Editorial: conflict and crisis settings: promoting sexual and reproductive rights. Reprod Health Matters. 2008;16(31):4-9. https://doi.org/10.1016/S0968-8080(08)31380-9. | Global | Conflict and emergency | Qualitative |
| Pillai VK, Gupta R. Reproductive rights approach to reproductive health in developing countries. Glob Public Health. 2011;4(1). http://dx.doi.org/10.3402/gha.v4i0.8423. | Global |  | Quantitative |
| Pillai VP, Wang Y-CP, Maleku AP. Women, war, and reproductive health in developing countries. Soc Work Health Care. 2017;56(1):28-44. http://dx.doi.org/10.1080/00981389.2016.1240134. | Global | Conflict | Mixed |
| Reichenbach L, Roseman M. Reproductive health and human rights: the way forward. Philadelphia; University of Pennsylvania Press; 2011. | Global |  | Qualitative |
| Reilly N. Linking local and global feminist advocacy: framing women's rights as human rights in the Republic of Ireland. Womens Stud Int Forum. 2007;30(2):114-33. https://doi.org/10.1016/j.wsif.2007.01.004. | Ireland |  | Qualitative |
| Rizvi N, Nishtar S. Pakistan's health policy: appropriateness and relevance to women's health needs. Health Policy. 2008;88(2-3):269-81. https://doi.org/10.1016/j.healthpol.2008.03.011. | Pakistan |  | Quantitative |
| Robinson F. Discourses of motherhood and women’s health: maternal thinking as feminist politics. JIPT. 2014;10(1):94-108. https://doi.org/10.1177/1755088213507189. | Global |  | Qualitative |
| Robinson M. Where human rights begin: health, sexuality, and women in the new millennium. New Brunswick; Rutgers University Press; 2005. | Global |  | Qualitative |
| Round Up. Round Up: The law, the courts, and sexual and reproductive rights. Reprod Health Matters. 2014;22(44):222-32. https://doi.org/10.1016/S0968-8080(14)44816-X. | Global |  | Qualitative |
| Sen G. Sexual and reproductive health and rights in the post-2015 development agenda. Glob Public Health. 2014;9(6):599-606. https://doi.org/10.1080/17441692.2014.917197. | Global |  | Qualitative |
| Sen G, Govender V. Sexual and reproductive health and rights in changing health systems. Glob Public Health. 2015;10(2):228-42. https://doi.org/10.1080/17441692.2014.986161. | Global |  | Qualitative |
| Sen G, Östlin P. Gender inequity in health: why it exists and how we can change it. Glob Public Health. 2008;(3(S1)):1-12. https://doi.org/10.1080/17441690801900795. | Global |  | Qualitative |
| Shaw D. Understanding the relevance of sexual and reproductive rights to professional responsibilities. J Obstet Gynaecol Can. 2004;26(12):1095-6. https://doi.org/10.1016/S1701-2163(16)30438-8. | Global |  | Qualitative |
| Shaw D. Women's right to health and the Millennium Development Goals: promoting partnerships to improve access. Int J Gynaecol Obstet. 2006;94(3):207-15. https://doi.org/10.1016/j.ijgo.2006.04.029. | Global |  | Qualitative |
| Simon-Kumar R. Neo-liberal development and reproductive health in India: the making of the personal and the political. Indian J Gend Stud. 2007;14(3):355-85. https://doi.org/10.1177/097152150701400301. | India |  | Qualitative |
| Smith SA. Sexual and reproductive health of Micronesians: a systematic review of the literature. Asia Pac J Public Health. 2013;25(1):7-18. https://doi.org/10.1177/1010539512458951. | Micronesia |  | Qualitative |
| Snow RC, Laski L, Mutumba M. Sexual and reproductive health: progress and outstanding needs. Glob Public Health. 2015;10(2):149-73. https://doi.org/10.1080/17441692.2014.986178. | Global |  | Mixed |
| Solinger R. Pregnancy and power: a short history of reproductive politics in America. New York; NYU Press; 2007. | United States |  | Qualitative |
| Spain D. Constructive feminism: women's spaces and women's rights in the American city. New York; Cornell University Press; 2016. | United States |  | Qualitative |
| Starrs AM, Ezeh AC, Barker G, Basu A, Bertrand JT, Blum R, et al. Accelerate progress—sexual and reproductive health and rights for all: report of the Guttmacher–Lancet Commission. Lancet. 2018;391(10140):2642-92. http://doi.org/10.1016/S0140-6736(18)30293-9. | Global |  | Mixed |
| Steans J, Ahmadi V. Negotiating the politics of gender and rights: some reflections on the status of women's human rights at ‘Beijing Plus Ten’. Glob Soc. 2005;19(3):227-45. https://doi.org/10.1080/13600820500135353. | Global |  | Qualitative |
| Stotland NL. Reproductive rights and women’s mental health. Psychiatr Clin North Am. 2017;40(2):335-50. https://doi.org/10.1016/j.psc.2017.01.010. | United States | Mental health | Qualitative |
| Telo SV, Witt RR. Sexual and reproductive health: team competences in primary health care services. Cien Saude Colet. 2018;23(11):3481-90. https://doi.org/10.1590/1413-812320182311.20962016. | Global |  | Mixed |
| Thanenthiran S. Twenty years and counting: taking the lessons learned from ICPD to move the sexual and reproductive health and rights agenda forward. Glob Public Health. 2014;9(6):669-77. http://dx.doi.org/10.1080/17441692.2014.920893. | Global |  | Qualitative |
| Thomas TN, Gausman J, Lattof SR, Wegner MN, Kearns AD, Langer A. Improved maternal health since the ICPD: 20 years of progress. Contraception. 2014;90(6, Supplement):S32-S8. https://doi.org/10.1016/j.contraception.2014.06.026. | Global |  | Qualitative |
| UN Population Fund (UNFPA). Rights into action, UNFPA implements human rights-based approach. New York: UNFPA; 2005. https://www.unfpa.org/sites/default/files/pub-pdf/rights_action.pdf. Accessed 14 September 2020. | Global |  | Qualitative |
| UNESCO, Joint United Nations Programme on HIV/AIDS, United Nations Population Fund, UN WOMEN, WHO, UNICEF. International technical guidance on sexuality education: an evidence-informed approach. Geneva: UNESCO Publishing; 2018. https://www.unfpa.org/sites/default/files/pub-pdf/ITGSE.pdf. Accessed 14 September 2020. | Global | Sexuality education | Qualitative |
| United Nations. Reproductive rights are human rights: A handbook for national human rights institutions. Copenhagen: UNFPA, The Danish Institute for Human Rights, United Nations Human Rights Office of the High Commissioner; 2014. https://www.unfpa.org/publications/reproductive-rights-are-human-rights. Accessed 13 January 2022. | Global |  | Qualitative |
| United Nations Population Fund. UNFPA operational guidance for comprehensive sexuality education: A focus on human rights and gender. New York: UNFPA; 2014. https://www.unfpa.org/sites/default/files/pub-pdf/UNFPA_OperationalGuidance_WEB3_0.pdf. Accessed 20 September 2020. | Global | Sexuality education | Qualitative |
| Vaidya S. Women with disability and reproductive rights: deconstructing discourses. Soc Change. 2015;45(4):517-33. https://doi.org/10.1177/0049085715602787. | India | Disability | Qualitative |
| Wielding S, Flynn B. Does service integration improve the sexual and reproductive healthcare of women living with HIV? Int J STD AIDS. 2015;27(12):1063-5. https://doi.org/10.1177/0956462415606341. | Scotland | HIV/AIDS | Quantitative |
| Yamin AE. From ideals to tools: applying human rights to maternal health. PLoS Med. 2013;10(11):e1001546. https://doi.org/10.1371/journal.pmed.1001546. | Global |  | Qualitative |
| Yamin AE, Boulanger VM. Why global goals and indicators matter: the experience of sexual and reproductive health and rights in the Millennium Development Goals. J Hum Dev Capab. 2014;15(2-3):218-31. http://dx.doi.org/10.1080/19452829.2014.896322. | Global |  | Qualitative |
