# Supplementary material for: The visibility of breastfeeding as a sexual and reproductive health right: a review of the relevant literature
Source: Int Breastfeed J. 2022 Mar 5;17:18. doi: 10.1186/s13006-022-00457-w (PMC8897768; doi:10.1186/s13006-022-00457-w)
Supplement: Supplementary file 2 — Additional file 2: Gender responsive budgeting literature review sample. This file includes the citations for each publication included in the sample for the review of the gender responsive budgeting literature. It also includes a brief note of each article’s geographical focus, sub-context and method. [file 13006_2022_457_MOESM2_ESM.docx]

*Gender responsive budgeting literature review sample*

| Sample publications | Geographical focus | Sub-context | Method |
| --- | --- | --- | --- |
| Addabbo T, Lanzi D, Picchio A. Gender Budgets: A Capability Approach. J Hum Dev Capab. 2010;11(4):479-501. http://doi.org/10.1080/19452829.2010.520900. | Global |  | Qualitative |
| Adeyeye MM, Akinbami CAO. Women and gender budgeting: Nigeria's policy alternative. Gender and Behaviour. 2010;8(1):2703-23. https://doi.org/10.4314/gab.v8i1.54688. | Nigeria |  | Qualitative |
| Alston M. Gender mainstreaming and climate change. Women’s Stud Int Forum. 2014;47:287-94. https://doi.org/10.1016/j.wsif.2013.01.016. | Global | Climate change | Qualitative |
| Asian Development Bank. Gender responsive budgeting in Vietnam: gender equality in transport. Ha Noi: Asian Development Bank Institute; 2019. https://www.adb.org/publications/budgeting-viet-nam-gender-equality-transport. Accessed 3 September 2020. | Vietnam | Transport | Qualitative |
| Avdagic M, Hujic F. Gender Responsive Budgeting as Smart Economics: A Comparative Analysis between Bosnia and Herzegovina and Republic of Macedonia. Journal of Economic and Social Studies. 2012;2(2):197-216. http://dx.doi.org/10.14706/JECOSS11229. | Bosnia and Herzegovina and Republic of Macedonia |  | Qualitative |
| Bakker I. Connecting Women's Human Rights to Public Resources in Canada. Can Woman Stud. 2018;33(1-2):71-7. | Canada |  | Qualitative |
| Bamanyaki P. Evaluating effects of local-level outside government gender budget initiatives in maternal health: an application of theory-based evaluation, process tracing and a quasi experiment in Kabale, Uganda. Antwerp: University of Antwerp; 2016. Accessed 4 September 2020. | Uganda | Maternal health | Qualitative |
| Bedford K. Care and the 53rd Commission on the Status of Women: a transformative policy space? Reprod Health Matters. 2011;19(38):197-207. https://doi.org/10.1016/S0968-8080(11)38576-X. | Global | Care | Qualitative |
| Bojičić-Dželilović V, Hozić AA. Taxing for inequalities: gender budgeting in the Western Balkans. Rev Int Polit Econ. 2020:1-25. http://doi.org/10.1080/09692290.2019.1702572. | Western Balkans |  | Qualitative |
| Botlhale E. Gender-responsive budgeting: The case for Botswana. Dev South Afr. 2011;28(1):61-74. http://doi.org/10.1080/0376835X.2011.545170. | Botswana |  | Qualitative |
| Budlender D. Budget call circular and gender budget statements in the Asia Pacific: a review. New Delhi: UN Women; 2016. https://asiapacific.unwomen.org/en/digital-library/publications/2016/05/budget-call-circulars-and-gender-budget-statements-in-the-asia-pacific. Accessed 13 January 2022. | Asia Pacific |  | Qualitative |
| Cagatay N. Gender Budgets and Beyond: Feminist Fiscal Policy in the Context of Globalisation. Gend Dev. 2003;11(1):15-24. https://doi.org/10.1080/741954249. | Global |  | Qualitative |
| Costa M. Gender Responsive Budgeting in Fragile States: The Case of Timor-Leste, 1 edn. Milton; Taylor & Francis Group; 2017. | Timor-Leste |  | Qualitative |
| Dey J, Dutta S. Gender responsive budgeting in India: trends and analysis. Int J Soc Sci Res. 2014;3(4):495-509. https://doi.org/10.5958/2321-5771.2014.00024.6. | India |  | Qualitative |
| Downes R, von Trapp L, Nicol S. Gender budgeting in OECD countries. OECD J Budg. 2017;16(3):1-37. http://dx.doi.org/10.1787/16812336. | OECD |  | Qualitative |
| Gill R, Stewart DE. Relevance of Gender-Sensitive Policies and General Health Indicators to Compare the Status of South Asian Women’s Health. Women's Health Issues. 2011;21(1):12-8. https://doi.org/10.1016/j.whi.2010.10.003. | South Asia |  | Mixed |
| Himmelweit S. Making policymakers more gender aware: experiences and reflections from the Women's budget group in the United Kingdom. J Women Politics Policy. 2005;27(1-2):109-21. https://doi.org/10.1300/J501v27n01_07. | United Kingdom |  | Qualitative |
| Holvoet N, Inberg L. Gender Responsive Budgeting and the Aid Effectiveness Agenda: Experiences from Mozambique. J Int Women's Stud. 2014;15(2):61-79. https://vc.bridgew.edu/jiws/vol15/iss2/5/. | Mozambique |  | Qualitative |
| IMF. Gender budgeting in G7 countries. Washington, DC: International Monetary Fund; 2017. https://www.imf.org/en/Publications/Policy-Papers/Issues/2017/05/12/pp041917gender-budgeting-in-g7-countries. Accessed 10 October 2020. | G7 |  | Qualitative |
| Kapungu R. The Zimbabwe gender budgeting and women's empowerment programme. Agenda: Empowering Women for Gender Equity. 2008;(78):68-78. https://doi.org/10.1080/10130950.2008.9674985. | Zimbabwe |  | Qualitative |
| Khalifa R, Scarparo S. Gender responsive budgeting: a tool for gender equality. Crit Perspect Account. 2020:102183-96. https://doi.org/10.1016/j.cpa.2020.102183. | Global |  | Qualitative |
| Khan Z, Burn Ne. Financing for gender equality: realising women's rights through gender responsive budgeting. New York; Springer Nature, Palgrave Macmillan; 2017. | Global |  | Qualitative |
| Lahey K. Australian tax-transfer policies and taxing for gender equality | Australia |  | Qualitative |
| Lahey KA. Women, substantive equality, and fiscal policy: gender-based analysis of taxes, benefits, and budgets. Can J Women Law. 2010;22(1):27-106. http://dx.doi.org/10.1353/jwl.2010.0007. | Canada |  | Qualitative |
| Mahadevia D, Bhatia N, Sebastian R. Gender responsive budgeting for Indian cities: the case of Bhopal and Pune. Environ Urban Asia. 2019;10(1):44-62. http://doi.org/10.1177/0975425318821806. | India |  | Mixed |
| Manyeruke C, Hamausw S. Feminisation of gender budgeting: an uphill task for Zimbabwe. Eastern Africa Social Science Research Review. 2013;29(1):77-105. http://doi.org/10.1353/eas.2013.0002. | Zimbabwe |  | Qualitative |
| Misra SN, Ghadai SK. Feminism, budgeting and gender justice. J Educ Prac. 2017;8(10):149-54. | India |  | Qualitative |
| Morrissey S. Implicit gender bias in GST systems. NZ J Tax L & Policy | New Zealand |  | Qualitative |
| Nakray K. Gender budgeting and public policy: the challenges to operationalising gender justice in India. Policy Polit. 2015;43(4):561-77. http://doi.org/10.1332/030557314X13914333479951. | India |  | Qualitative |
| Nallari R, Griffith B. Gender and macroeconomic policy. Washington, DC: World Bank; 2011. https://openknowledge.worldbank.org/handle/10986/2256. Accessed 20 October 2020. | Global |  | Qualitative |
| Pacoy EP. Reducing gender gaps through gender-responsive budgeting in Davao City, Philippines. In: Millennium Development Goals and Community Initiatives in the Asia Pacific*.* Edited by Singh A, Gonzalez ET, Thomson SBe. New Delhi: Springer India; 2013: 97-106. | Philippines |  | Qualitative |
| Patel V. Women and inclusive growth. Indian Econ J. 2011;58(4):164-74. https://doi.org/10.1177/0019466220110410. | India |  | Qualitative |
| Rai SM, Brown BD, Ruwanpura KN. SDG 8: Decent work and economic growth – A gendered analysis. World Dev. 2019;113:368-80. https://doi.org/10.1016/j.worlddev.2018.09.006. | Global | Decent work | Qualitative |
| Rajneesh S. Gender budgeting to gender mainstreaming. Indian J Public Adm. 2008;54(4):904-7. https://doi.org/10.1177/0019556120080408. | India |  | Qualitative |
| Rao S. Reforms with a female face: gender, liberalization, and economic policy in Andhra Pradesh, India. World Dev. 2008;36(7):1213-32. https://doi.org/10.1016/j.worlddev.2007.06.020. | India |  | Quantitative |
| Rubin MM, Bartle JR. Integrating gender into government budgets: a new perspective. Public Adm Rev. 2005;65(3):259-72. http:doi.org/10.1111/j.1540-6210.2005.00452.x. | Global |  | Qualitative |
| Shash FG, Forden CL. Gender equality in a time of change: Gender mainstreaming after Egypt's Arab Spring. Womens Stud Int Forum. 2016;56:74-82. https://doi.org/10.1016/j.wsif.2015.12.006. | Egypt |  | Qualitative |
| Singh D. The gender dimensions of expenditure in India: some policy issues and concerns. Product. 2018;59(2):111-20. | India |  | Mixed |
| Sodani PR, Sharma S. Gender responsive budgeting. J Health Manag. 2008;10(2):227-40. http://doi.org/10.1177/097206340801000205. | India, Pakistan, Nepal, Sri Lanka |  | Qualitative |
| Stotsky GJ, Zaman A. The influence of gender budgeting in Indian states on gender inequality and fiscal spending. Washington, DC: International Monetary Fund; 2016. https://www.imf.org/external/pubs/ft/wp/2016/wp16227.pdf. Accessed 4 October 2020. | India |  | Quantitative |
| Stotsky JG. Gender budgeting : fiscal context and current outcomes. Washington, DC: International Monetary Fund; 2016. https://www.imf.org/en/Publications/WP/Issues/2016/12/31/Gender-Budgeting-Fiscal-Context-and-Current-Outcomes-44132. Accessed 4 October 2020. | Global |  | Qualitative |
| Stotsky JG. Using fiscal policy and public financial management to promote gender equality: international perspectives. Milton; Taylor & Francis Group; 2020. | Global |  | Qualitative |
| Stotsky JG, Kolovich L, Kebhaj S. Sub-Saharan Africa: a survey of gender budgeting efforts. Washington, DC: International Monetary Fund; 2016. https://www.imf.org/external/pubs/ft/wp/2016/wp16152.pdf. Accessed 4 October 2020. | Sub-Saharan Africa |  | Mixed |
| Zakirova V. Gender inequality in Russia: the perspective of participatory gender budgeting. Reprod Health Matters. 2014;22(44):202-12. http://dx.doi.org/10.1016/S0968-8080(14)44806-7. | Russia |  | Qualitative |
|  |  |  |  |
