# Supplementary material for: The visibility of breastfeeding as a sexual and reproductive health right: a review of the relevant literature
Source: Int Breastfeed J. 2022 Mar 5;17:18. doi: 10.1186/s13006-022-00457-w (PMC8897768; doi:10.1186/s13006-022-00457-w)
Supplement: Supplementary file 3 — Additional file 3: References to breastfeeding in the sexual and reproductive health rights literature review. This file includes the citations for the publications which referred to breastfeeding in the sexual and reproductive health rights literature review. These publications are separated into, firstly, those that contained references to breastfeeding which were not deemed to be about the protection, promotion or support of breastfeeding, and secondly publications that did contain references to the protection, promotion or support of breastfeeding. A summary of the context of the references to breastfeeding are included for each publication. [file 13006_2022_457_MOESM3_ESM.docx]

*References to breastfeeding in the sexual and reproductive rights literature review*

| References to breastfeeding not deemed to be about the protection, promotion or support of breastfeeding | | |
| --- | --- | --- |
| Source | Context of reference | |
| Alzate MM. The role of sexual and reproductive rights in social work practice. Affilia. 2009;24(2):108-19. https://doi.org/10.1177%2F0886109909331695. | Mentions ‘drug use during pregnancy and lactation’ and ‘breastfeeding practices’ in a table listing ‘some sexual and reproductive health concerns in social work practice’ (Page 111) | |
| Hill BJ. Reproductive rights as health care rights. Colum J Gender & L. 2008;18(2):501-50. https://doi.org/10.7916/cjgl.v18i2.2570. | “In particular, the government was concerned that the efficacy of the treatment could be undermined by multiple social, economic, and cultural factors, such as… the expense of providing infant formula to HIV-positive mothers, the difficulty of persuading women to substitute that formula for breastfeeding, and the absence of clean water in certain parts of the country, which would make formula feeding riskier” in relation to a South African Constitutional Court case dealing with access to an antiretroviral drug (Page 19) | |
| Nair S, Sexton S, Kirbat P. A decade after Cairo: women's health in a free market economy. Indian J Gend Stud. 2006;13(2):171-93. https://doi.org/10.1177/097152150601300203. | Notes that barrier methods of contraception ‘have no adverse impact on breastfeeding’ but receive limited research funding, promotion and distribution as part of population programs (Page 184) | |
| Solinger R. Pregnancy and power: a short history of reproductive politics in America. New York; NYU Press; 2007. | Enslaved women “were also forced to perform the reproductive labor of wet-nursing, that is, nursing baby born to another woman” [Page 37]. On the control of nursing enslaved women, “mothers who left the fields to nurse their children were threatened with the whip. Going further, the owner tried to establish a rigid three-times-a-day schedule for infant feeding” (Page 39). Enslaved women were pressured “to wean their babies early’ in order to enhance fertility and hence increase slave labor. (Page 36). Cites historian’s assessment that “relatively poor nutritional content and quantity of the slave mother’s milk probably triggered early weaning and shorter birth intervals” [Page 36]. Describes studies that showed ‘women in the second half of the eighteenth century were likely to rely on breastfeeding to limit their fertility, a strategy that proved itself “a feeble method”’ [Page 56]. On the forced migration of Mexican women lead to ‘high rates of women who cannot follow traditional practices of breastfeeding’ (Page 114) | |
| Vaidya S. Women with disability and reproductive rights: deconstructing discourses. Soc Change. 2015;45(4):517-33. https://doi.org/10.1177/0049085715602787. | “Motherhood is a social role with tremendous cultural and ideological heft; the bringing forth of new life from one’s body through the processes of conception, gestation and birthing, sustenance through lactation, nurturing and the practices of care are surrounded by webs of meaning that shape and transform the embodied experience of mothering.” (Page 519) | |
| References to the protection, promotion and support of breastfeeding | | |
| Source | | Context of reference |
| Alburo-Cañete KZK. Bodies at risk: “managing” sexuality and reproduction in the aftermath of disaster in the Philippines. Gend Technol Dev. 2014;18(1):33-51. https://doi.org/10.1177/0971852413515356. | “To address reproductive health needs, government agencies and other groups… monitored and recorded the number of pregnant and lactating women” (Page 38)  “…in the early months after the disaster, pregnant and lactating mothers comprised over 5 percent of the affected population” (Page 40)  Management strategies included “putting up of bulletin boards in camp command centres to account for the number of …, lactating mothers still in the shelters” (Page 44)  “Other SRH services provided in the camps came in the form of “parenting” seminars, providing vitamins to pregnant and lactating women, ante-natal check-ups, and promoting proper hygiene” (Page 45)  The authors describe an understanding of sexual and reproductive health to include caring for newborns and infants, maintaining sanitation, healthcare facility provision, curbing maternal deaths, the prevention of STIs, and planning pregnancy; this is a broader definition of sexual and reproductive health apparent in the broader literature, and is more responsive to the inclusion of breastfeeding issues. (Page 45) | |
| Alzate MM. The sexual and reproductive rights of internally displaced women: the embodiment of Colombia's crisis. Disasters. 2008;32(1):131-48. https://doi.org/10.1111/j.1467-7717.2007.01031.x. | Citing Colombian legislation, ‘Law 100 of 1993 established that all citizens would receive health care services by 2001 regardless of their working status or economic means—a target that was not achieved. The minimum required health package (POS), includes reproductive health services mainly for prenatal care, delivery/post-delivery, and breastfeeding complications.’ The paper does not go on to specifically analyse breastfeeding, but notes that law 100 did not guarantee the provision of these services due to lack of government funding, overwhelmed hospital facilities, and payment issues in regard to internally displaced persons. (Page 139) | |
| Chaudhuri S. A life course model of human rights realization, female empowerment, and gender inequality in India. World Dev. 2013;52:55-70. http://doi.org/10.1016/j.worlddev.2013.07.001. | Notes children’s rights to access breastfeeding [Page 56], cites a study on the gender gap in breastfeeding leading to female infants being less likely to be breastfed (page 58), includes breastfeeding as an indicator of whether a child is realising her right to health with detailed analysis (Pages 60-63, 67), notes the WHO Guidelines on infant feeding practices (Page 60) | |
| Danielsson M, Sundström K. Chapter 6: reproductive health. Scand J Public Health. 2006;34(67_suppl):147-64. http://doi.org/10.1080/14034950600677204. | “maternity health care includes… , encouraging breast feeding,….” (Page 159) | |
| Galtry J. Strengthening the human rights framework to protect breastfeeding: a focus on CEDAW. Int Breastfeed J. 2015;10:29. https://doi.org/10.1186/s13006-015-0054-5. | Article centred on breastfeeding and human rights | |
| Gupta GR, Oomman N, Grown C, Conn K, Hawkes S, Shawar YR, et al. Gender equality and gender norms: framing the opportunities for health. Lancet. 2019;393(10190):2550-62. https://doi.org/10.1016/S0140-6736(19)30651-8. | In relation to efforts to hold corporate actors accountable, “one such collective effort in Vietnam shows how the government, with the help of CSO Alive and Thrive and UNICEF, banned advertising of breast milk substitutes and, along with other efforts (mass-media campaign, counselling, new policy on maternity leave), increased rates of exclusive breastfeeding, ensuring nutrition for infants during the first 6 months of life” (Page 2557) | |
| Haslegrave M. Integrating sexual and reproductive rights into the medical curriculum. Best Pract Res Clin Obstet Gynaecol. 2006;20(3):433-45. https://doi.org/10.1016/j.bpobgyn.2006.01.002. | Includes Article 7.6 of the 1994 ICPD Programme of Action, whereby the definition of the term "reproductive health services" includes ‘… education and services for prenatal care, safe delivery, and postnatal care, especially breastfeeding and infant and women’s health care’ ([United Nations, 1994](#_heading=h.1d96cc0)) (Page 433) | |
| Koenen KC, Lincoln A, Appleton A. Women's status and child well-being: A state-level analysis. Soc Sci Med. 2006;63(12):2999-3012. https://doi.org/10.1016/j.socscimed.2006.07.013. | “If women have poor access to pre- and post-natal medical care, they are unlikely to receive information on proper nutrition during pregnancy and on the benefits of breastfeeding, both of which have direct consequences for child out-comes such as birthweight” (Page 3000)  No further analysis, and breastfeeding is not included as an indicator to measure women’s reproductive rights. Indicators included a number of abortion access scores, insurance coverage laws for contraception and infertility treatments, same-sex couple adoption, and mandatory sex education | |
| Mehta S. The AIDS pandemic: A catalyst for women's rights. Int J Gynaecol Obstet. 2006;94(3):317-24. https://doi.org/10.1016/j.ijgo.2006.04.020. | Pregnant women who receive a positive HIV result should receive ‘…counselling and treatment for preventing transmission to their baby, including advice on breastfeeding and alternatives..’ (Page 320)  Notes that the majority of children who become infected with HIV do so ‘during their mother’s pregnancy, labor and delivery, or as a result of breastfeeding’ (Page 321)  ‘HIV transmission from mother to child can be prevented through… alternatives to breastfeeding.’ (Page 321)  ‘Even if a baby is born negative, there is a risk of transmission from breastfeeding’ (Page 321)  ‘Counselling on breastfeeding and other options is an important aspect of postpartum care, and healthcare workers need regular training so they can offer up-to-date information. Most importantly, if replacement feeding is not possible, WHO recommends exclusive breastfeeding during the first months of life’ (Page 322)  Notes insufficient research on ‘the implications of ART in breast milk’ (Page 323) | |
| Ngwena CG, Brookman-Amissah E, Skuster P. Human rights advances in women’s reproductive health in Africa. Int J Gynaecol Obstet. 2015;129(2):184-7. https://doi.org/10.1016/j.ijgo.2015.02.001. | Cites article 14 of the Protocol to the African Charter on Human and Peoples’ Rights on the Right of Women in Africa ([African Union, 2003](#_heading=h.26in1rg)), which includes breastfeeding health and nutritional services under article 14(2)(b). The article does not go on to discuss breastfeeding specifically. (Page 185) | |
| Reichenbach L, Roseman M. Reproductive health and human rights: the way forward. Philadelphia; University of Pennsylvania Press; 2011. | “… promotion of breastfeeding and growth monitoring of infants….were able to reduce the rates of infant and child death and disease’ (Page 93)  “The agendas of the sexual and reproductive health and HIV communities are increasingly overlapping, with such shared concerns as gender dynamics, human rights, sexuality, pregnancy, childbirth, and breastfeeding.” (Page 136) | |
| Rizvi N, Nishtar S. Pakistan's health policy: appropriateness and relevance to women's health needs. Health Policy. 2008;88(2-3):269-81. https://doi.org/10.1016/j.healthpol.2008.03.011. | Mentions calorie intake and iron deficiency of lactating mothers as part of a table of women’s health indicators (Page 273) | |
| Shaw D. Understanding the relevance of sexual and reproductive rights to professional responsibilities. J Obstet Gynaecol Can. 2004;26(12):1095-6. https://doi.org/10.1016/S1701-2163(16)30438-8. | Includes Article 7.6 of the 1994 ICPD Programme of Action, as above ([United Nations, 1994](#_heading=h.1d96cc0)) (Page 1095) | |
| Shaw D. Women's right to health and the Millennium Development Goals: promoting partnerships to improve access. Int J Gynaecol Obstet. 2006;94(3):207-15. https://doi.org/10.1016/j.ijgo.2006.04.029. | “Breastfeeding can be part of the solution to multiple goals” especially in eradicating poverty and hunger (Page 208)  Mentions a case of a young woman denied termination of pregnancy and required to breastfeed her anencephalic infant in Peru (Page 213) | |
| Starrs AM, Ezeh AC, Barker G, Basu A, Bertrand JT, Blum R, et al. Accelerate progress—sexual and reproductive health and rights for all: report of the Guttmacher–Lancet Commission. Lancet. 2018;391(10140):2642-92. http://doi.org/10.1016/S0140-6736(18)30293-9. | Comments in relation to antiretroviral therapy to pregnant and breastfeeding women to virtually eliminate transmission of HIV from mother to child in parts of the world. (Page 2656)  Routine check-ups and support for breastfeeding is specifically mentioned as part of postnatal care for a continuum of quality care, as well as information about breastfeeding and STIs. (Page 2659)  Counselling on lactational amenorrhea mentioned as part of information on contraceptive methods to be given to women post-partum. (Page 2677) | |
| UNESCO, Joint United Nations Programme on HIV/AIDS, United Nations Population Fund, UN WOMEN, WHO, UNICEF. International technical guidance on sexuality education: an evidence-informed approach. Geneva: UNESCO Publishing; 2018. https://www.unfpa.org/sites/default/files/pub-pdf/ITGSE.pdf. Accessed 14 September 2020. | Learning objectives for 9-12 years in relation to sexual and reproductive health education: ‘learners will be able to… explain that with support from family, the community, services and treatment, women living with HIV can be healthy and deliver and breastfeed children who are HIV free’ (Page 77)  Notes that breastfeeding is a method of HIV transmission (Page 79) | |
| United Nations. Reproductive rights are human rights: A handbook for national human rights institutions. Copenhagen: UNFPA, The Danish Institute for Human Rights, United Nations Human Rights Office of the High Commissioner; 2014. https://www.unfpa.org/publications/reproductive-rights-are-human-rights. Accessed 13 January 2022. | Recommends to National Human Rights Institutions (NHRIs) in the context of leading by example through internal policies, ‘breastfeeding mothers should be able to plan their work in such a way as to enable breastfeeding according to WHO advice (exclusive breastfeeding for the first six months of the child’s life). (Page 37)  Children’s Breastfeeding Rights: Notes in relation to the right to the highest attainable standard of health (Article 12 of the International Covenant on Economic, Social and Cultural Rights), that promotion of breastfeeding has been encouraged by the Committee on the Rights of the Child [Page 112, (Page 180). Cites Article 14 of the African Charter on the Rights and Welfare of the Child which ‘obliges states to take measures to reduce infant and child mortality , to ensure appropriate care for expectant and nursing mothers, and to ensure the knowledge by all sectors of society of, among other things, the advantages of breastfeeding’. (Page 194)  Women’s Breastfeeding Rights Instruments:  Cites Article 12 of CEDAW guaranteeing women ‘*appropriate services in connection with pregnancy, confinement and the post-natal period, granting free services where necessary, as well as adequate nutrition during pregnancy and lactation*’ (Page 109). References the CEDAW General Recommendation No. 26 – Women migrant workers, 2008: citing that countries of destinations have a duty to ensure “pregnant and breastfeeding mothers [in detention] have access to appropriate services” [Page 149]. Cites article 14 of the Protocol to the African Charter on Human and Peoples’ Rights on the Right of Women in Africa ([African Union, 2003](#_heading=h.26in1rg)), which includes breastfeeding health and nutritional services under article 14(2)(b), as well as Article 24 which provides for women In distress that nursing women be provided with an environment suitable to their condition and treated with dignity. (Page 193)  Cites a recent initiative, the Campaign on Accelerated Reduction of Maternal Mortality in Africa (CARMMA), which in Sierra Leone introduced free treatment and medicines for pregnant women, lactating mothers and children under five at all government health facilities (Page 199)  References a right to breastfeed case in Portugal (Page 221) | |
| UN Population Fund (UNFPA). Rights into action, UNFPA implements human rights-based approach. New York: UNFPA; 2005. https://www.unfpa.org/sites/default/files/pub-pdf/rights_action.pdf. Accessed 14 September 2020. | Cites article 12 of the Convention on the Elimination of Discrimination Against Women (CEDAW) which calls on states to provide *‘women appropriate services in connection with pregnancy, confinement and the post-natal period, granting free services where necessary, as well as adequate nutrition during pregnancy and lactation*’ (["Convention on the Elimination of All Forms of Discrimination Against Women," 1979](#_heading=h.3o7alnk)). No further analysis on breastfeeding is included in the report (Page 20) | |
