# Supplementary material for: The visibility of breastfeeding as a sexual and reproductive health right: a review of the relevant literature
Source: Int Breastfeed J. 2022 Mar 5;17:18. doi: 10.1186/s13006-022-00457-w (PMC8897768; doi:10.1186/s13006-022-00457-w)
Supplement: Supplementary file 4 — Additional file 4: References to breastfeeding in the gender responsive budgeting literature review. This file includes the citations for the publications which referred to breastfeeding in the gender responsive budgeting literature review. These publications are separated into, firstly, those that contained references to breastfeeding which were not deemed to be about the protection, promotion or support of breastfeeding, and secondly publications that did contain references to the protection, promotion or support of breastfeeding. A summary of the context of the references to breastfeeding are included for each publication. [file 13006_2022_457_MOESM4_ESM.docx]

*References to breastfeeding in the gender responsive budgeting literature review*

| References to breastfeeding not deemed to be about the protection, promotion and support of breastfeeding | |
| --- | --- |
| Source | Context of reference |
| Patel V. Women and inclusive growth. Indian Econ J. 2011;58(4):164-74. https://doi.org/10.1177/0019466220110410. | Mentions nursing as an example of women’s work burden in the unpaid care economy (Page 165) |
| Dey J, Dutta S. Gender responsive budgeting in India: trends and analysis. Int J Soc Sci Res. 2014;3(4):495-509. https://doi.org/10.5958/2321-5771.2014.00024.6. | Mentions provisions for supplementary feeding of nursing mothers as part of the Third, Fourth and Interim five-year plans (1961-74) (Page 499) |
| Misra SN, Ghadai SK. Feminism, budgeting and gender justice. J Educ Prac. 2017;8(10):149-54. | Mentions the Anganwadi program to provide an integrated health, supplementary nutrition and education to nursing mothers, amongst others (Page 152) |
| Rajneesh S. Gender budgeting to gender mainstreaming. Indian J Public Adm. 2008;54(4):904-7. https://doi.org/10.1177/0019556120080408. | Mentions the supply of nutritious food to nursing mothers through Anganwadi centres (a type of rural childcare centre in India) (Page 906) |
| References to the protection, promotion or support of breastfeeding | |
| Source | Context of reference |
| Budlender D. Budget call circular and gender budget statements in the Asia Pacific: a review. New Delhi: UN Women; 2016. https://asiapacific.unwomen.org/en/digital-library/publications/2016/05/budget-call-circulars-and-gender-budget-statements-in-the-asia-pacific. Accessed 13 January 2022. | Extract from Indonesian budget statement includes a recommendation that airports add a nursery room in the departure and arrival terminals (Page 18) |
|  |  |
